# Probiotics and Prebiotics in Cardiovascular Diseases

**DOI:** 10.3390/nu15173686

**Published:** 2023-08-23

**Authors:** Miguel Romero, Juan Duarte

**Affiliations:** 1 Department of Pharmacology, School of Pharmacy and Center for Biomedical Research (CIBM), University of Granada, 18071 Granada, Spain; 2 Instituto de Investigación Biosanitaria de Granada (ibs.GRANADA), 18012 Granada, Spain; 3 Centro de Investigación Biomédica en Red en Enfermedades Cardiovasculares (CIBER-CV), Instituto de Salud Carlos III, 28029 Madrid, Spain

This Special Issue, titled “Probiotics and Prebiotics in Cardiovascular Diseases”, encompasses two comprehensive review articles examining the potential of gut-microbiota-targeted reprogramming interventions designed to prevent the onset and progression of cardiovascular diseases. These interventions aim to restore structural and functional changes in gut microbiota while maintaining immune system homeostasis. Additionally, four original articles explore the positive effects of diverse prebiotics, probiotic strains, and antibiotic strategies on the pathogenic mechanisms involved in hypercholesterolemia, obesity, and hypertension. These interventions encompass the modulation of inflammatory and immune responses, improvements in vascular alterations, the enhancement of intestinal barrier function, and the beneficial effects on gut dysbiosis.

Cardiovascular diseases (CVDs) encompass a group of abnormalities affecting the cardiovascular system, including coronary heart disease, cerebrovascular disease, and peripheral vascular disease. Despite notable advances in pharmacological treatment, the prevalence of CVDs continues to rise, making them a leading cause of global morbidity and mortality [1,2]. While several drugs are available for managing CVDs, their effectiveness is often limited, and may be accompanied by significant side effects [3]. As a result, novel and safe strategies, such as lifestyle modifications and nutritional approaches, are imperative [4,5].

Remarkably, growing evidence suggests that gut microbiota in early life influences the developmental origins of CVD in adult offspring [6]. The article by Hsu and colleagues in this Special Issue [7] reviews the effectiveness of gut-microbiota-targeted interventions, including prebiotics, probiotics, and postbiotics, as potential reprogramming strategies to prevent the developmental origins of CVDs based on animal studies. However, uncertainties remain about the effectiveness and long-term effects of these interventions in humans, necessitating further research and clinical trials to validate the findings and establish optimal intervention approaches.

Moreover, the onset and progression of CVDs are associated with various risk factors, such as chronic kidney disease, obesity, type 2 diabetes mellitus, insulin resistance, dyslipidemia, atherosclerosis, and hypertension [8]. Over the last few decades, substantial interest has centered around the role of gut microbiota in the development of CVDs. Emerging evidence indicates that changes in the composition and function of the intestinal microbiota, referred to as dysbiosis, play a significant role in the pathogenesis of CVDs through diverse mechanisms, including inflammatory responses and autoimmune disorders. As a result, there is growing support for the targeting of gut microbiota through dietary or pharmacological interventions as a promising therapeutic strategy for managing cardiovascular risk [9].

For this Special Issue, Wu and Chiou conducted a review article exploring the potential beneficial effects of prebiotics and probiotics on cardiovascular diseases (CVDs), with a specific focus on coronary heart disease and stroke [10]. The authors provide updated insights into how prebiotics and probiotics can protect against CVDs by restoring structural and functional changes in gut microbiota and maintaining immune homeostasis. The review highlights several protective effects, including improved gut barrier function, the rebalancing of dysbiotic gut microbiota, a reduction in cardiovascular markers (such as cholesterol levels and high blood pressure), the attenuation of oxidative stress, the effects on other tissues through neurotransmitter production and immunomodulation via the gut–brain axis, and the enhancement of anti-inflammatory responses. Additionally, short-chain fatty acids, the main metabolites produced by gut microbiota from prebiotics, play a crucial role in preventing CVDs. However, the underlying mechanisms of their protective effects are complex and require further investigation through clinical evidence.

Furthermore, functional foods containing bioactive compounds, prebiotics, and/or probiotics have shown promise in preventing various cardio-metabolic disorders, including obesity, hyperlipidemia, and hypertension [11]. Among these bioactive compounds, organosulfur compounds from vegetables like *Allium*, such as propyl propane thiosulfinate (PTS) and propyl-propane thiosulfonate (PTSO), have garnered interest for their bioactive properties [12,13]. These properties may explain the potential protective effects of PTS on metabolic health. A study in this Special Issue by Liébana-Garcia and colleagues [14] investigated the potential anti-obesogenic effects of PTS in a murine model of diet-induced obesity. The authors demonstrated that the oral administration of PTS in a dose-dependent manner prevented the weight gain and metabolic dysfunction caused by a hypercaloric diet. Moreover, the higher dose of PTS improved glucose and hepatic homeostasis, modulated lipid metabolism, and increased the thermogenic capacity of brown adipose tissue. These effects led to reduced inflammation, increased thermogenesis, and preserved hepatic and intestinal homeostasis. Notably, PTS did not significantly alter the microbial ecosystem, suggesting that its protective effects may be attributed to its immunomodulatory and anti-inflammatory properties. While this study offers preclinical evidence supporting the protective impact of PTS against obesity, further research is necessary to validate these findings in human studies.

Hypercholesterolemia and hypertension are two crucial risk factors for CVDs [15]. Recent studies in both animal models and humans have indicated a strong connection between shifts in gut microbiota and its metabolites with hypertension and hypercholesterolemia [16,17]. As a result, probiotic interventions that regulate the composition and diversity of the intestinal microbiota hold promise for effectively treating these disorders. In this context, two papers in this Special Issue explore the influence of probiotics on these diseases. Firstly, Yang and colleagues [18] examined the cholesterol-lowering effects of two probiotic strains (*Enterococcus faecium* strain 132 and *Lactobacillus paracasei* strain 201) isolated from human feces in an animal model of hypercholesterolemia induced by a high-cholesterol diet. The results indicated that both strains reduced liver inflammation, improved the lipid profile by regulating the gene expression related to cholesterol metabolism and reduced fat accumulation. Additionally, both strains modulated the gut microbiota by decreasing the abundance of certain bacterial families related to hypercholesterolemia and increasing the levels of acetic acid and propionic acid in feces. These findings suggest that the *E. faecium* strain 132 and *L. paracasei* strain 201 may alleviate hypercholesterolemia in rats and have potential applications as functional foods for managing high cholesterol levels. Secondly, in another article of this monograph [19], the authors evaluated the anti-hypertensive effects of *Lactobacillus fermentum* CECT5716 and *Bifidobacterium breve* CECT7263 in a murine model of systemic lupus erythematosus induced by Toll-Like receptor 7 (TLR7) activation. Hypertension is a primary risk factor for the development of renal and CVDs, which are leading causes of mortality among systemic lupus erythematosus patients [20]. Previous studies have shown that TLR7 activation is associated with hypertension, endothelial dysfunction and gut dysbiosis. This effect is attributed, at least in part, to increased vascular inflammation and oxidative stress resulting from immune dysregulation, specifically affecting Th17 cell polarization [21,22]. In this study, de la Visitación and colleagues [19], demonstrated for the first time that chronic treatment with the probiotics *Lactobacillus fermentum* CECT5716 or *Bifidobacterium breve* CECT7263 prevented hypertension and endothelial dysfunction in a mouse lupus model induced by TLR-7 activation. These results suggest that changes in gut microbiota may influence the mechanisms involved in the development of hypertension after TLR7 activation, through a reduction in SLE activity and a decrease in vascular oxidative stress and inflammation, possibly due to the reduction in Th17 and increase in Treg populations in mesenteric lymph nodes, thereby restoring the Th17/Treg balance in vascular tissues.

Finally, the modulation of the gut microbiome through antibiotic strategies could potentially impact the prevalence and origin of hypertension. Notably, the broad-spectrum tetracycline antibiotic doxycycline has shown to attenuate blood pressure increase in different animal models of hypertension and to improve various aspects of vascular health [23,24,25]. In another article within this monograph [26], researchers investigated whether doxycycline could prevent cardiovascular pathology and reduce hypertension in deoxycorticosterone acetate (DOCA)-salt rats, a renin-independent model of hypertension. The study revealed that chronic doxycycline treatment also prevented blood pressure increase and improved endothelial function in this low renin model of hypertension. These findings suggest that doxycycline can influence the gut microbiota and improve intestinal barrier function, owing to its direct impact on the gut microbiota, as well as its anti-inflammatory and immunomodulatory properties. Consequently, this leads to a decrease in the endotoxemia and vascular dysfunction associated with DOCA-salt hypertension. These effects are linked to a reduction in NADPH oxidase-dependent ROS production and an increase in Treg infiltration and IL-10 within the vascular wall, contributing to the restoration of immune dysregulation.

In summary, modulating the gut microbiota through dietary or pharmacological interventions holds promise as a therapeutic target for managing cardiovascular-related diseases. The studies featured in this Special Issue play a critical role in advancing our understanding of the role of gut microbiota in the origin and development of CVDs. Moreover, these findings provide crucial insights into the potential positive effects of prebiotics, probiotic strains, and antibiotic strategies on the pathogenic mechanisms involved in hypercholesterolemia, obesity, and hypertension. These effects include the modulation of inflammatory and immune responses, an improvement in vascular alterations, the enhancement of the intestinal barrier function, and a beneficial impact on gut dysbiosis. However, the precise mechanisms underlying the gut microbiota’s health-promoting role in CVD prevention remain largely unclear, necessitating further research.
